# Control of bleeding from a recurrent tumor at a uretero‐ileal anastomosis by electrocoagulation via an ileal conduit

**DOI:** 10.1002/iju5.12613

**Published:** 2023-07-31

**Authors:** Rikuto Yasujima, Yasukazu Nakanishi, Kohei Hirose, Yosuke Umino, Naoya Okubo, Madoka Kataoka, Shugo Yajima, Hitoshi Masuda

**Affiliations:** ^1^ Department of Urology National Cancer Center Hospital East Chiba Japan

## Abstract

**Introduction:**

Bladder cancer is characterized by spatial and temporal recurrence in the urinary tract. We describe a case of recurrence at a uretero‐ileal anastomosis after radical cystectomy and nephroureterectomy. It was difficult to control bleeding from the tumor, but hemostasis was achieved.

**Case presentation:**

A 73‐year‐old man with a history of radical cystectomy and reconstruction of the ileal conduit and right nephroureterectomy was diagnosed with recurrence at the uretero‐ileal anastomosis site. Bleeding from the tumor could not be controlled by flexible gastrointestinal endoscopy. The patient underwent coagulation via an ileal conduit approach using a rigid scope and bipolar electrocautery, which is usually a modality for transurethral resection.

**Conclusion:**

This is the first report in which a modality normally used for transurethral resection was used to control bleeding in a patient with an ileal conduit. This application is useful in cases open surgery or additional irradiation might be difficult.

**Keynote message**

We report a case in which we controlled the bleeding from a recurrence tumor at anastomosis using only electrocoagulation via an ileal conduit. In situations where open surgery or additional irradiation are considered difficult, this method might be an option.

**Abbreviations & Acronyms**
CT: computed tomography
NMIBC: non‐muscle‐invasive bladder cancer
RC: radical cystectomy
TURBT: transurethral resection of bladder tumor
UC: urothelial carcinoma

## Introduction

Bladder cancer is characterized by spatial and temporal recurrence in the urinary tract. After RC, the rate of recurrence in the urinary tract is 4%–10%.^1^ and this is the most common site of late recurrence (after 3 years postoperatively).^2^, ^3^, ^4^ In this case, we experienced recurrence at a uretero‐ileal anastomosis 5 years after RC and ileal conduit creation, resulting in severe acute anemia due to bleeding from the tumor. However, we successfully controlled the bleeding using only electrocoagulation via an ileal conduit.

## Case

The patient was a 73‐year‐old man with a past medical history of significant diabetes, asthma, hypertension, and percutaneous coronary intervention for coronary stenosis, and he took aspirin. He had a history of three TURBT with pathologically confirmed high‐risk NMIBC followed by RC and reconstruction of an ileal conduit by Wallace plate technique at age 69. Pathological examination revealed a UC, low grade (G2), pTa, and the bilateral margins were negative. Furthermore, he underwent right open nephroureterectomy due to right lower ureteral recurrence at age 72. During the operation, because part of the ureter‐ileal anastomosis was firmly adhered, the ureter was dissected and cut as far as possible distally. Adjuvant chemotherapy was not administered based on the pathological findings of UC, high grade (G2), pT2, and absence of lympho‐vascular invasion and a resection margin.

**Fig. 1:**
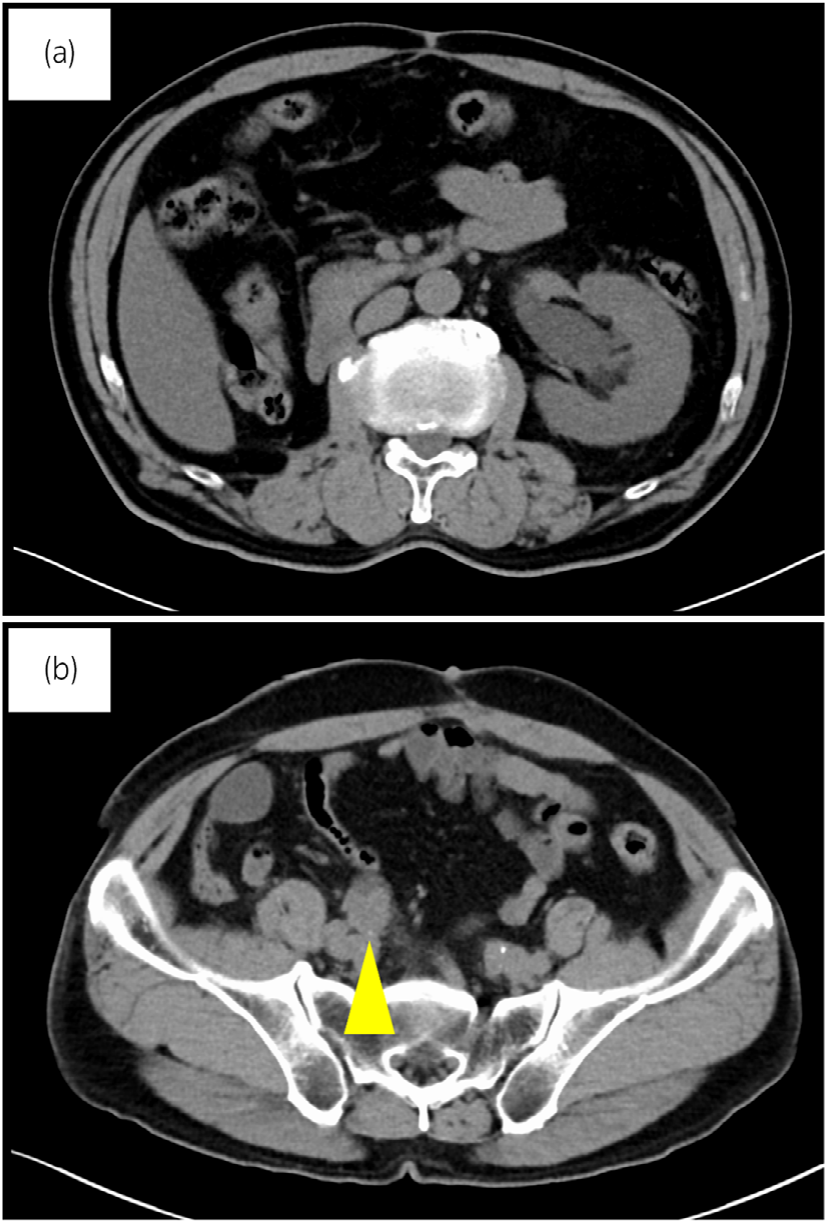
Axial CT showing (a) left hydronephrosis and acute pyelonephritis and (b) a recurrent mass at the left uretero‐ileal anastomosis (yellow arrowhead).

One year after the right nephroureterectomy, CT showed left hydronephrosis and acute pyelonephritis. A recurrent mass at the left uretero‐ileal anastomosis was noted (Fig. 1). After left percutaneous nephrostomy placement and antibiotic therapy, irradiation therapy of 50 Gy/25 fractions was administered, because complete surgical resection was deemed difficult due to the high degree of adhesions associated with previous surgeries.

Six months after irradiation, CT showed multiple lung metastases, lumbar bone metastases, and aortic lymph node metastases, and then systemic chemotherapy with gemcitabine/carboplatin was planned.

**Fig. 2:**
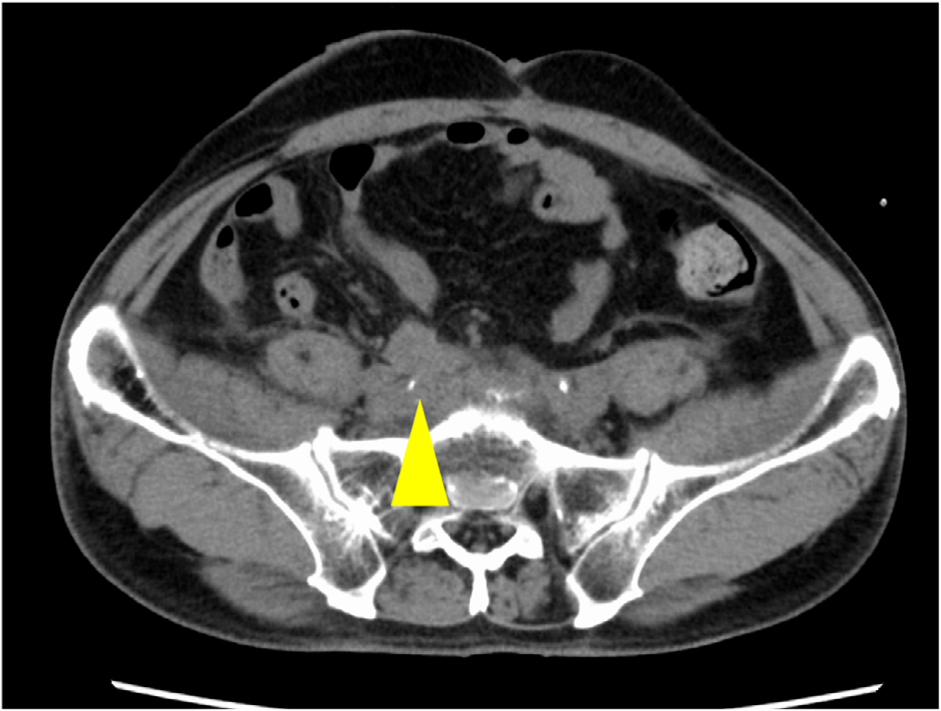
Re‐enlargement of the recurrent mass at the uretero‐ileal anastomosis revealed by axial CT (yellow arrowhead).

**Fig. 3:**
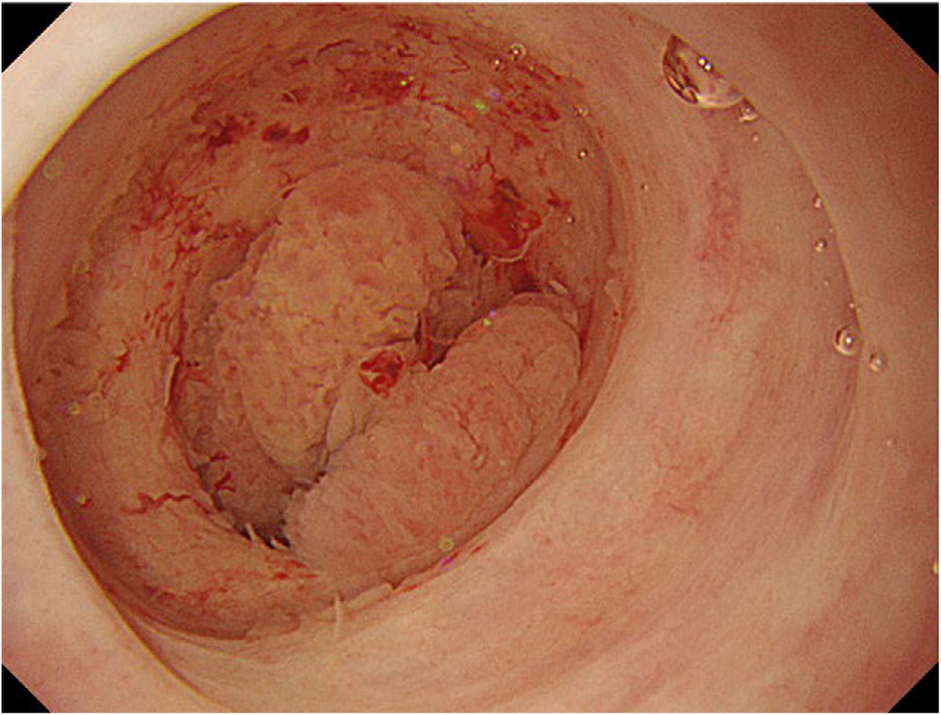
The tumor visualized by a flexible gastrointestinal endoscope. A papillary tumor was observed, but the source of bleeding could not be identified.

However, at the time of chemotherapy induction, the patient developed severe anemia with a Hb level of 5.6 g/dL due to ileal conduit bleeding. CT showed re‐enlargement of the recurrent mass at the uretero‐ileal anastomosis (Fig. 2). The patient had multiple metastases and a history of radiation therapy, so we chose palliative therapy. We considered the location of the bleeding site and decided that direct hemostasis was preferable. Because of persistent bleeding after blood transfusion, flexible gastrointestinal endoscopic hemostasis was initially attempted. However, a tumor was observed, but the source of bleeding could not be identified (Fig. 3). So, we could not perform any invasive procedures, only observation. Due to persistent bleeding afterward, we performed coagulation via an ileal conduit approach using a rigid scope and bipolar electrocautery, which is usually used as a modality for TUR under general anesthesia. We inserted a rigid cystoscope through the ileal conduit opening in the supine position. Removal of the internal coagula as much as possible allowed us to identify the anastomosis and recurrent mass. When the surface of the recurrent mass was scraped with a loop electrode, the source of the hemorrhage could be determined. We performed coagulation and hemostasis using rollerball and loop electrodes. Finally, we applied oxidized regenerated cellulose (Surgicel®, Johnson & Johnson, America) to the surface of the residual mass (Surgery time: 122 min).

Postoperatively, systemic chemotherapy was introduced, and no rebleeding was observed during the 8‐month follow‐up.

## Discussion

We performed nephroureterectomy on the first recurrence of the right lower ureter. No tumor infiltration was observed microscopically at the resection margins, although we were unable to remove the uretero‐ileal anastomosis. Usually, however, the anastomosis should be removed using a technique such as segmental uretero‐ileal conduit resection, as reported by Zeng *et al*.^5^

In this case, the second recurrence may be a recurrence of the residual ureter at the time of nephroureterectomy. In a report of 19 cases by Zattoni *et al*., the anastomosis was the most common site of recurrence after ileal conduit creation, and surgical resection was the treatment of choice in nearly 80% of cases,^6^ but in this case of anastomotic recurrence, we selected radiation therapy due to the history of multiple open ureteral surgeries and the high degree of adhesion already present at the time of the previous surgery.

When dealing with bleeding in patients with a history of treatment like this one, hemostatic maneuvers similar to those used for gastrointestinal bleeding are an option. Ultimately, we chose a rigid cystoscope, which has two advantages. First, because an ileal conduit is a totally incontinent urinary diversion, the intraductal pressure does not increase even during the procedure requiring irrigation. As a result, rupture or anastomotic leakage can be prevented. Second, the high perfusion efficiency provides a relatively good field of view. This method could be one of the options for dealing with cases such as the present case.

## Conclusion

We experienced a case of controlled bleeding from a UC recurring at the uretero‐ileal anastomosis, which was treated by electrocoagulation via an ileal conduit. Although this appears to be a safe procedure, further study is needed for cases in which palliative treatment must be selected.

## Author contributions

Rikuto Yasujima: Conceptualization; writing – original draft. Yasukazu Nakanishi: Conceptualization; supervision; writing – review and editing. Kohei Hirose: Supervision; writing – review and editing. Yosuke Umino: Supervision; writing – review and editing. Naoya Okubo: Supervision; writing – review and editing. Madoka Kataoka: Supervision; writing – review and editing. Shugo Yajima: Conceptualization; supervision; writing – review and editing. Hitoshi Masuda: Conceptualization; supervision; writing – review and editing.

## Conflict of interest

The authors declare no conflict of interest.

## Approval of the research protocol by an Institutional Reviewer Board

Not applicable.

## Informed consent

Informed consent was obtained from the patient for the release of this case report.

## Registry and the Registration No. of the study/trial

Not applicable.
